# Resilience in Family Caregivers of Adults With Autism Spectrum Disorder: An Integrative Review of the Literature

**DOI:** 10.1093/geroni/igab046.2986

**Published:** 2021-12-17

**Authors:** Daphne Chakurian

**Affiliations:** University of Missouri - Columbia, Roseville, California, United States

## Abstract

Care of adults with Autism Spectrum Disorder (ASD) is a public health priority and costs are projected to be 549 billion US dollars by 2025. Middle and older adult FCGs of adults with ASD often provide lifelong care, experience chronic stress, consequently, are at risk of poor mental health and QOL. An integrative review examined factors associated with resilience in studies of middle and older adult FCGs of adults with ASD. A comprehensive literature search found 10 reports of 8 studies published in peer-reviewed scholarly journals before October 13, 2020. Studies and/or reports of factors associated with resilience in middle and older adult FCGs of adults with ASD were examined using PRISMA, and quality checklists. Some 340 articles met search criteria, 14 were fully reviewed, and 10 were included. Findings suggest FCGs of adults with ASD show capacity for resilience consistent with research on FCGs of children with ASD significant chronic stress. A broad range of resilience factors were studied, and resilience was associated with positive social support, higher QOL, self-efficacy, and problem and meaning-focused coping styles. There is a dearth of research on middle and older adult FCGs of adults with ASD. Increased reporting of social determinants of health and participation of underrepresented groups is needed. Future research must address FGC heterogeneity and specify theoretically grounded conceptual and operational definitions of resilience. Identifying resilience factors is necessary for intervention studies to enhance resilience.

